# Risk factors for COVID-19-related death, hospitalization and intensive care: a population-wide study of all inhabitants in Stockholm

**DOI:** 10.1007/s10654-021-00840-7

**Published:** 2022-01-27

**Authors:** Maria-Pia Hergens, Max Bell, Per Haglund, Johan Sundström, Erik Lampa, Joanna Nederby-Öhd, Maria Rotzén Östlund, Thomas Cars

**Affiliations:** 1 Department of Medicine Karolinska Solna, Unit of Infectious Diseases, Karolinska Institutet, Stockholm, Sweden; 2 Department of Communicable Disease Control and Prevention, Stockholm Region, Sweden; 3 Section of Anaesthesiology and Intensive Care Medicine, Department of Physiology and Pharmacology, Karolinska Institutet, Stockholm, Sweden; 4 Department of Perioperative Medicine and Intensive Care (PMI), Karolinska University Hospital, Solna, Stockholm, Sweden; 5 Department of Health Care Analysis and Statistics, Public Healthcare Services Committee, Stockholm Region, Sweden; 6 Department of Medical Sciences, Uppsala University, Uppsala, Sweden; 7 The George Institute for Global Health, University of New South Wales, Sydney, NSW Australia; 8 Department of Global Public Health, Karolinska Institutet, Stockholm, Sweden

**Keywords:** Covid 19, Risk factors, Epidemiology

## Abstract

Since the beginning of the Covid-19 pandemic, the scientific community has explored determinants of Covid 19 disease severity. However, the majority of studies are based on in-hospital patients with high risk of collider- or selection bias. The present investigation details risk factors associated with overall mortality, hospitalization and intensive care unit (ICU) admission in Covid-19 infections, with complete population coverage and high-resolution data on patient characteristics and comorbid conditions This population-based observational study comprises all residents 18 years and older in Stockholm Region—1.8 million inhabitants—using the real-time Covid-19 monitoring framework. The observation period lasted between March 1 to December 31, 2020. Hazard ratios (HR) for risk factors of Covid-19 disease severity were assessed using Cox proportional hazard models. In total, 3322 deaths, 11,508 hospitalizations and 1423 ICU-admissions related to Covid-19 occurred during the study period. Kidney failure, diabetes and obesity increased risk of mortality and so did heart failure and ischemic heart disease. However, atrial fibrillation and hypertension did not. Risk of hospitalization follow a similar pattern, whereas admission to intensive care differs; triage processes where clearly present as certain co-morbid conditions were associated with lower ICU admission. Observed differences in risk of mortality and hospitalization among patients with Covid 19 raise important questions about potentially protective comedication which will be further addressed using the real-time Covid-19 monitoring framework.

**Supplementary Information:**

The online version contains supplementary material available at 10.1007/s10654-021-00840-7.

## Introduction

The novel severe acute respiratory syndrome coronavirus 2 (SARS-CoV-2) causes Coronavirus disease 2019 (Covid-19). Since 31 December 2019 until today (31 December 2020) over 100 million cases and over 2.4 million deaths have been reported [1] and it continues to be a large global health threat. The Stockholm Region in Sweden was hit hard by the pandemic with a surge in cases at the end of March and beginning of April 2020 (Supplementary Figs. S1 and S2).

Studies of risk factors for adverse Covid-19 outcomes, for patients already infected are plentiful and have focused on hypertension, obesity, diabetes and cardiovascular comorbidity [2–4]. A systematic review of 61 cohort studies with 31,089 patients hospitalized with Covid-19 reported increased risks of mortality with underlying chronic kidney disease, cardiovascular disease, cerebrovascular disease, chronic obstructive pulmonary disease, hypertension, malignancy, diabetes and immunodeficiency [5]. Such observations can be useful for triaging hospitalized patients, but do not predict risks of adverse outcomes in the general population. This potential collider bias has been discussed in several papers [6, 7].

Further, multiple investigations use severity of disease and intensive care unit (ICU) admission as an outcome [5]. This is problematic; we lack consensus on how severity of Covid-19 is defined and ICU admissions are affected by ICU resources. Lastly, complicating matters is the fact that certain risk factors could be linked to the *exposure for* SARS-CoV-2; examples are obesity and smoking, associated with lower socioeconomic status [8] and overrepresentation in jobs where working from home is uncommon whilst frequent person encounters is common.

In this population-wide study comprising all inhabitants 18 years and older in the Stockholm Region, we aimed at investigating risk factors for severe Covid-19 while addressing the above-mentioned issues. We primarily investigated factors of risk for overall mortality and hospitalization; secondarily, we analyzed risk factors for ICU admission with Covid-19. Uniquely, this study uses information from the real-time Covid-19 monitoring framework in Region Stockholm.

## Methods

This was a population-based observational study comprising all residents 18 years and older in Region Stockholm.

### Data sources

Region Stockholm manages a central healthcare utilization data warehouse (called VAL). This includes comprehensive information from inpatient, hospital outpatient, and primary care, including information on healthcare utilization, consultations and diagnoses at an individual level. VAL also contains demographic information on patient age, sex, migration status, death and information from the Population Register [9] as well as socio‐economic status, defined using Mosaic data described elsewhere [10]. The coverage in VAL for inpatient care is over 99% [11] and for diagnostic coding, validity is estimated to 85–95%, depending on the diagnosis [12]. SmiNet is the national electronic surveillance system for the reporting of communicable diseases [13]. Since February 1, 2020, it is mandatory for all Swedish laboratories to report findings of Covid-19 to SmiNet. Information from VAL and SmiNet were linked using the unique personal identity number given to each Swedish citizen [14].

### Study populations

The primary study population consisted of all individuals 18 years and older, residing in Stockholm county on March 1, 2020 based on data from the Population Register [9]. In a secondary study population, we excluded all individuals permanently staying in elderly care facilities, since they by routine stayed at the facility and were treated for covid-19 there and hence did not contribute risk to be admitted to hospitals or ICU, see flowchart in Supplementary Fig. S3). A Covid-19 case is confirmed by Nucleic Acid Detection of SARS Betacorona Group Specific Gene Fragment or isolation of SARS-CoV-2 [15]. To confirm Covid-19 cases the diagnostic method of polymerase chain reaction (PCR) was set up in January 2020; it is the method used to detect active infection of Covid-19 in Sweden.

### Outcomes

The primary outcome was mortality with confirmed Covid-19 infection. The secondary outcome was hospitalization with confirmed Covid-19 infection. The tertiary outcome was admission to intensive care (ICU) with confirmed Covid-19 infection.

### Follow-up

The observation period was March 1, 2020 to December 31, 2020. Follow-up began on March 1, 2020 and ended at loss to follow-up (emigration from the Stockholm Region), end of study (December 31, 2020), death (not for the primary outcome), or the outcomes.

### Risk factors

Comorbidities considered as potential risk factors in the study were extracted from VAL using ICD-10 codes recorded up to five years prior to the start of the study period (March 1, 2020). Codes recorded in inpatient, hospital outpatient, and primary care (in any diagnosis position) were included. The ICD-10 codes used to define potential risk factors are presented in Supplementary Table S1. The selection of risk factors in this study is based on the comorbidities presented in previous publications (mentioned in the introduction) as well as indications of high prevalence comorbidities observed during surveillance of the epidemiological data among hospitalized Covid-19 cases within the Stockholm Region.

### Statistical analyses

In order to identify bias-minimized models, causal diagrams were established for each potential risk factor (Supplementary Figs. S4–S10) using www.dagitty.net. The bias-minimized models are presented in Supplementary Table S2.

**Fig. 1:**
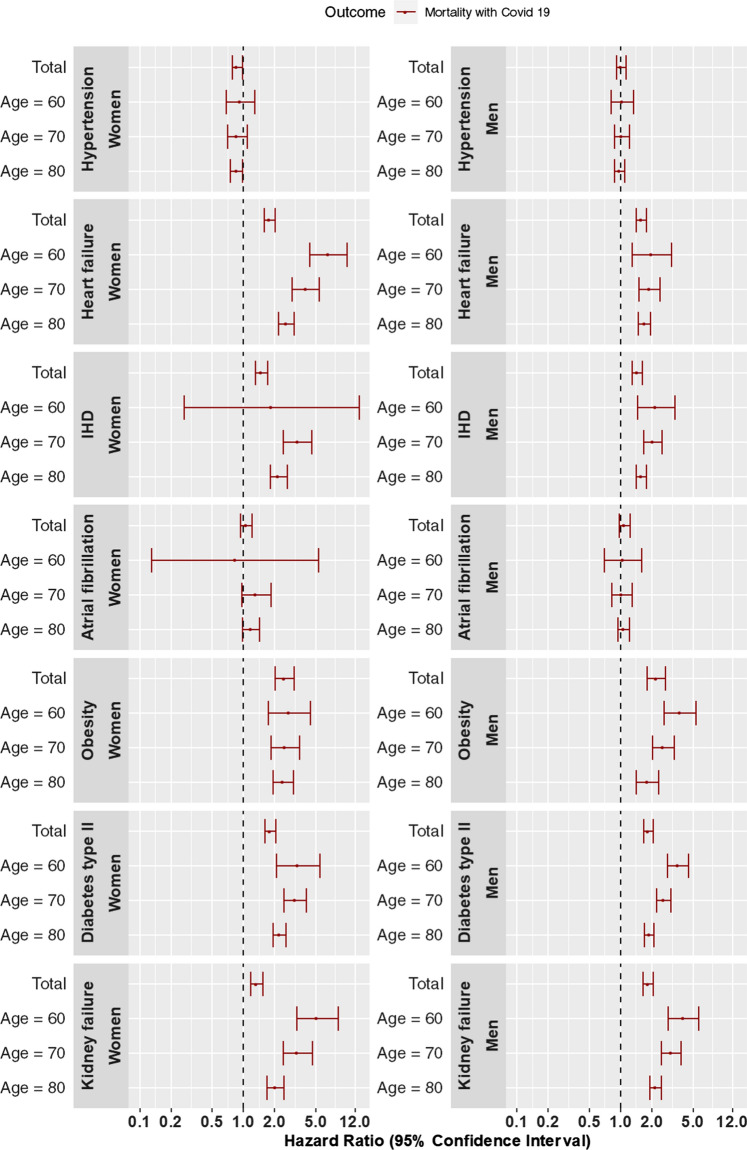
Associations of potential risk factors with the primary outcome (mortality with PCR-confirmed Covid-19). PCR, polymerase chain reaction

**Fig. 2:**
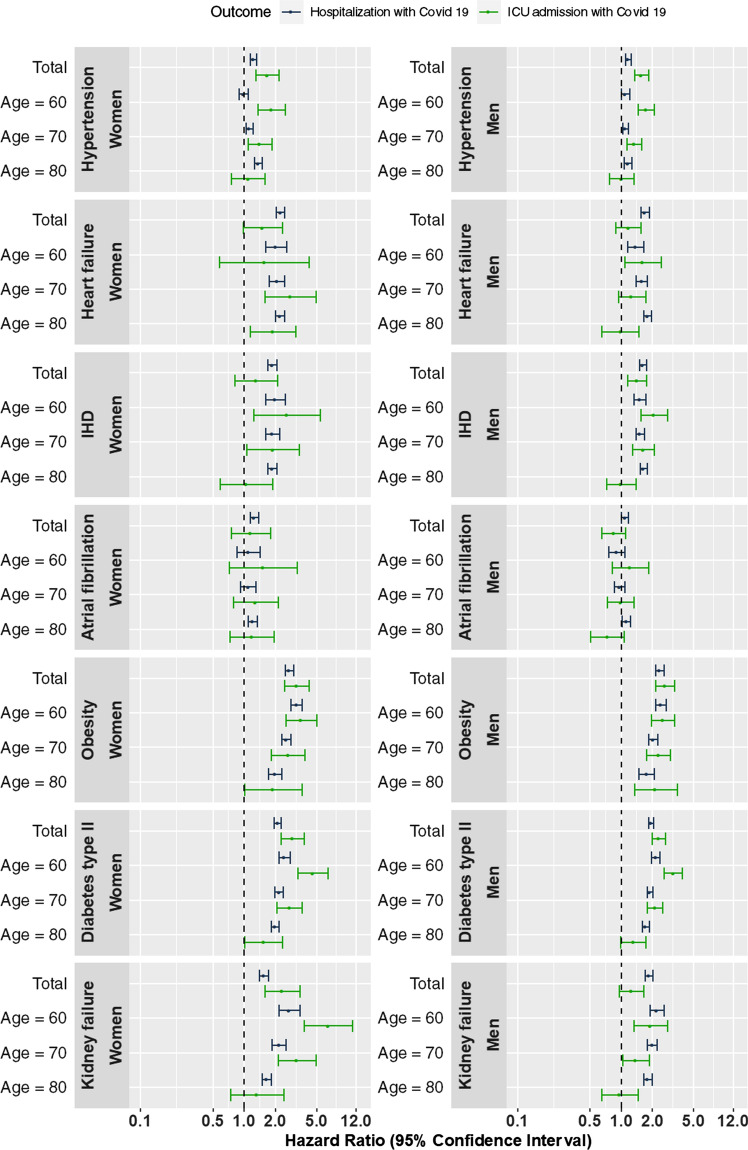
Associations of potential risk factors with the secondary (hospitalization with PCR-confirmed Covid-19) and tertiary outcomes (ICU admission with PCR-confirmed Covid-19). PCR, polymerase chain reaction

Associations between each potential risk factor and the different outcomes were assessed using Cox proportional hazards models. All models included a sex by other covariates interactions to obtain sex specific estimates and age was modeled using restricted cubic splines with knots at the 10^th^, 50^th^ and 90^th^ percentiles of the age distribution. Other covariates included in the models were guided by the causal diagrams (Supplementary Table S2). Socioeconomic status was included as a stratification variable, i.e., we allowed for different baseline hazards within each stratum. Two models were fitted for each risk factor/outcome pair; one where we assume additive effects of age and the risk factor within each sex and one where we allowed for an interaction to obtain age-specific estimates within each sex. Estimates from the first model is illustrated in the rows named Total in Figs. 1 and 2 whereas estimates for three different ages (60, 70, 80) from the second model follow in the rows below. The proportional hazards assumption was tested by testing the assumed independence of the scaled Schoenfeld residuals and time. All data management were done by MPH, TC, PH and EL, performed using SAS 9.4 (SAS Institute, Cary, US) and all analyses were made using the freely available statistical software R [16]version 3.6.0 with the rms add-on package [17].

### Ethical consideration

The study was approved by the Swedish Ethical Review Authority (Dnr: 2020-03,122). All data were analyzed in a pseudonymized format and confidentiality was maintained at all times.

## Results

In total 3,322 cases of Covid-19 deaths occurred during the study period with the vast majority of deaths occurring among those 80 years of age and older (68.4%) (Table 1). For the secondary outcome (hospitalization with Covid-19) the pattern was similar with a total of events during the study period, with the highest percentage of events in patients 80 years and older (25.4%) (Table 2). In contrast, intensive care admission (n = 1,423) was less common among patients over 80 years (n = 92, 6.5%) and highest among the age group 60–69 years (n = 430, 30.2%) (Table 3). Male sex was associated with higher mortality, hospital- and intensive care consumption (Tables 1, 2, 3). This was most clear for the tertiary outcome where more than 70% of the events occurred among the men (Table 3).

**Table 1:** Characteristics of the primary study population and rates of the primary outcome (mortality with PCR-confirmed Covid-19)

	Number of individuals (%)	Number of events (%)	Person years at risk (PYAR)	Events/1000 PYAR
Total	1,877,100 (100.0)	3322 (100.0)	1,550,459	2.14
Sex				
Female	943,598 (50.3)	1534 (46.2)	779,765	1.97
Male	933,502 (49.7)	1788 (53.8)	770,695	2.32
Age groups				
18–39 years	720,868 (38.4)	11 (0.3)	591,388	0.02
40–49 years	334,351 (17.8)	25 (0.8)	277,270	0.09
50–59 years	303,372 (16.2)	85 (2.6)	252,046	0.34
60–69 years	224,220 (11.9)	211 (6.4)	186,150	1.13
70–79 years	191,323 (10.2)	717 (21.6)	158,895	4.51
≥ 80 years	102,966 (5.5)	2273 (68.4)	84,710	26.83
Risk factors				
Hypertension	320,184 (17.1)	2199 (66.2)	265, 412	8.29
Heart failure	31,998 (1.7)	851 (25.6)	26,239	32.43
Ischemic heart disease	48,317 (2.6)	693 (20.9)	39,893	17.37
Atrial fibrillation	56,099 (3.0)	933 (28.1)	46,255	20.17
Obesity	63,344 (3.4)	190 (5.7)	52,505	3.62
Diabetes type II	96,632 (5.1)	913 (27.5)	79,963	11.42
Kidney failure	33,045 (1.8)	776 (23.4)	27,138	28.59

*PCR* polymerase chain reaction

The primary study population consists of all individuals 18 years and older residing in Stockholm County on March 1, 2020

**Table 2:** Characteristics of the secondary study population and rates of the secondary outcome (inpatient hospitalization with PCR* confirmed Covid-19)

	Number of individuals (%)	Number of events (%)	Person years at risk (PYAR)	Events/1000 PYAR
Total	1,861,772 (100.0)	11,508 (100.0)	1,531,200	7.51
Sex				
Female	933,339 (50.1)	4935 (42.9)	768,485	6.42
Male	928,433 (49.9)	6573 (57.1)	762,715	8.62
Age groups				
18–39 years	720, 863 (38.7)	1192 (10.4)	590,723	2.02
40–49 years	334,339 (18.0)	1172 (10.2)	276,675	4.24
50–59 years	303,293 (16.3)	1911 (16.6)	250,939	7.62
60–69 years	223,645 (12.0)	2105 (18.3)	184,401	11.42
70–79 years	188,312 (10.1)	2210 (19.2)	154,958	14.26
≥ 80 years	91,320 (4.9)	2918 (25.4)	73,504	39.70
Risk factors				
Hypertension	311,209 (16.7)	5452 (47.4)	254,821	21.40
Heart failure	29,059 (1.6)	1387 (12.1)	22,873	60.64
Ischemic heart disease	46,099 (2.5)	1409 (12.2)	37,224	37.85
Atrial fibrillation	52,350 (2.8)	1584 (13.8)	42,121	37.60
Obesity	63,043 (3.4)	1070 (9.3)	51,709	20.69
Diabetes type II	93,997 (5.0)	2582 (22.4)	76,406	33.79
Kidney failure	30,775 (1.7)	1293 (11.2)	24,415	52.96

*PCR* polymerase chain reaction

The secondary study population consists of all individuals 18 years and older residing in Stockholm County on March 1, 2020, excluding all individuals permanently staying in elderly care facilities

**Table 3:** Characteristics of the secondary study population and rates of the tertiary outcome (admission to Intensive Care Unit with PCR* confirmed Covid-19)

	Number of individuals (%)	Number of events (%)	Person years at risk (PYAR)	Events/1000 PYAR
Total	1,861,772 (100.0)	1423 (100.0)	1,534,880	0.93
Sex				
Female	933,339 (50.1)	402 (28.3)	770,149	0.52
Male	928,433 (49.9)	1021 (71.7)	764,731	1.34
Age groups				
18–39 years	720,863 (38.7)	103 (7.2)	591,234	0.17
40–49 years	334,339 (18.0)	140 (9.8)	277,123	0.51
50–59 years	303,293 (16.3)	343 (24.1)	251,635	1.36
60–69 years	223,645 (12.0)	430 (30.2)	185,087	2.32
70–79 years	188,312 (10.1)	315 (22.1)	155,570	2.02
≥ 80 years	91,320 (4.9)	92 (6.5)	74,232	1.24
Risk factors				
Hypertension	311,209 (16.7)	664 (46.7)	256,374	2.58
Heart failure	29,059 (1.6)	87 (6.1)	23,219	3.75
Ischemic heart disease	46,099 (2.5)	125 (8.8)	37,600	3.32
Atrial fibrillation	52,350 (2.8)	107 (7.5)	45,529	2.52
Obesity	63,043 (3.4)	159 (11.2)	52,066	3.05
Diabetes type II	93,997 (5.0)	355 (24.9)	77,145	4.60
Kidney failure	30,775 (1.7)	100 (7.0)	24,731	4.04

*PCR* polymerase chain reaction

The secondary study population consists of all individuals 18 years and older residing in Stockholm County on March 1, 2020, excluding all individuals permanently staying in elderly care facilities

### Risk factors for the primary outcome, mortality with Covid-19

Adjusted Hazard Ratios and 95% confidence intervals are shown in Supplementary Table S3 and Fig. 1. Heart failure, IHD, obesity, diabetes type II and kidney failure were associated with a higher risk of mortality with Covid-19 for both men and women whereas data did not support associations for hypertension and atrial fibrillation and Covid-19 related mortality. Furthermore, associations for all risk factors were similar for men and women with the exception of kidney failure where we observed a higher risk of mortality with Covid-19 for men (HR = 1.85, 95% CI: 1.65–2.08) than women (HR = 1.35, 95% CI: 1.18–1.55). For obesity, we observed an age-dependent association of mortality with Covid-19 with a lower risk at higher age.

### Risk factors for the secondary outcome, inpatient hospitalization with Covid-19

All risk factors were associated with hospitalization with Covid-19 (Fig. 2 and Supplementary Table S3). There was some evidence of an age-dependent association for hypertension where younger individuals were less likely to be admitted to hospital compared to older individuals, especially older women (*p*-value for three-way interaction between sex, age and hypertension < 0.001). Similar patterns were observed for heart failure and IHD where older individuals had a higher risk of being hospitalized than younger individual (*p*-value for interaction = 0.008 for heart failure and 0.016 for IHD). For obesity, diabetes type II and kidney failure we observed the opposite pattern with an increased risk for younger to be admitted to hospital hospitalized compared to older individuals (*p*-values for interaction < 0.001 for obesity and diabetes type II and < 0.001 for kidney failure). Data did support a three-way interaction (*p* < 0.001) between sex, age and obesity where younger women in particular had a higher risk (HR = 3.21, 95% CI: 2.84–3.62 at age 60; HR = 2.56, 95% CI: 2.31–2.84 at age 70) than younger men (HR = 2.42, 95% CI: 2.15–2.72 at age 60; HR = 2.05, 95% CI: 1.84–2.27 at age 70) of being hospitalized.

### Risk factors for the tertiary outcome, ICU admission with Covid-19

Hypertension, obesity and diabetes type II were all associated with a higher risk of ICU admission with Covid-19 in both men and women (Supplementary Table S3 and Fig. 2) whereas data did not support any association between atrial fibrillation on ICU admission with Covid-19. For kidney disease, there was some evidence of a sex-dependent association where women were more likely to be admitted to ICU compared to men (*p* = 0.002).

## Discussion

This cohort study of over 1.8 million people used the Region Stockholm real-time Covid-19 monitoring framework, allowing for detailed analysis of risk factors for Covid-19 related mortality, hospitalization and ICU-admission. The key findings include that across age groups and irrespective of sex, kidney failure, diabetes type II and obesity are associated with higher risk of mortality. In contrast, cardiovascular comorbidities diverge; heart failure and ischemic heart disease are risk factors for death, but atrial fibrillation and hypertension are not. Risks of hospitalization with regards to comorbid conditions follow a similar pattern, whereas admission to intensive care differs. Triage flow processes were clearly present as demonstrated by hazard ratios for heart failure, IHD and kidney failure; strongly associated with death and hospitalization but not with ICU admission. Ages 80 years and up accounted for two thirds of all Covid-19-related deaths in Stockholm.

Initial studies of risk factors have focused solely on patients with Covid-19. In hospitalized patients from Wuhan increasing age, higher illness severity score and increased d-dimer were associated with risk of in-hospital death [18]. A systematic review detailed impact of comorbidities on the disease course of Covid-19. It included 61 cohort studies of 31,089 patients. Cerebrovascular disease, chronic obstructive pulmonary disease, cardiovascular disease, hypertension, diabetes mellitus and malignancy were risk factors for poor clinical outcome in Covid-19 in that study [5]. As mentioned in the introduction, the two definitions of “poor clinical outcome” of Covid-19 across these studies were either severity of Covid-19 or admission to intensive care. Both outcomes lack rigid definitions. ICU admission is prone to regional or local variability as it is affected by medical tradition as well as ICU bed availability. Only 12 of these 61 investigations reported mortality. The differing findings between this systematic review and our findings, specifically that pulmonary disease and malignancy were not associated with mortality risk in our study, are likely explained by the fact that we used mortality as a hard endpoint while the above-mentioned review included multiple outcomes. Further, the cohorts included in that review run a high risk of collider bias [6, 7], which likely affects risk factors differentially.

The present investigation has an advantage: insight into the co-morbid burden for a study population comprising over 1.8 million individuals (see Tables 1, 2, 3). Similarly, the UK OpenSAFELY study had access to primary care records of over 17 million adults, linked to 10,926 Covid-19-related hospital deaths [3]. Increasing age, deprivation, being male; as well as diabetes, severe asthma, liver disease, kidney disease and multiple other comorbidities were associated to increased risk of dying of or with Covid-19. Using data from the real-time Covid-19 monitoring framework in Region Stockholm we have built a comprehensive overview on diagnosis from primary care, inpatient, and outpatient specialist care as well as information on conformed Covid-19 from a large, well-defined health care region. The present study differs from the UK study with regards to some key elements: firstly, we have complete coverage of all adult Stockholm residents. Secondly, we have data on both hospital- and out-of-hospital mortality and not only hospital mortality. Lastly, we report risk of hospitalization and ICU admission. This tertiary outcome, admission to intensive care, missing from the UK study, must however be analyzed with caution. Investigating co-morbid conditions as risk factors and their association to a soft outcome like ICU admission is always tricky, and more so during a pandemic. ICU admission is affected by ICU capacity. In a study from 2012, Sweden had 5.8 ICU beds/100,000 inhabitants as compared with the EU average of 11.5 [19]. Notably, the Stockholm ICU bed density is even lower at 4.2/100,000 [20]. Despite the fact that emergency changes to increase intensive care capacity were implemented [21] it was paramount to select patients with the highest chance of benefitting from ICU admission.

The results of the real-time Stockholm Covid-19 monitoring framework, combined with rigorous modeling, adjusting for age, sex and socioeconomic status using causal diagrams, show how comorbid conditions are or are not associated with risk of hospitalization or death. This study relies on the accuracy of diagnoses reported in medical records in which some might be missing or misclassified. However, the diagnostic validity of recorded diagnoses in Sweden in is generally high [12]. This allows for development of predictive models that in turn can be used for granular health care planning. Naturally, despite efforts to control for associations with certain co-morbidities and SARS-CoV-2 exposure and measures taken to minimize risk of bias, causality cannot be proven. Clearly, multiple observations in the present investigation indicate the need for rigorous and focused studies; are patients with atrial fibrillation protected by the fact that they almost always medicate with warfarin or novel oral anticoagulants? This question deserves further attention as several reports show that thrombotic events in hospitalized Covid-19 patients are common and dangerous [22]. Indeed, the in-hospital/in-ICU clinical practice has undergone rapid changes, with thrombotic awareness and increased use of anticoagulation. Our study sheds more—but not enough—light on hypertension; a non-significant risk factor for most age strata in our adjusted models. Interestingly, hypertension has been reported to have an inverse association with mortality among elderly UK patients [3] but was very frequent in both US and Chinese case series [23]. Focus could also be devoted to how kidney failure seemingly affects ICU patient selection in males and females.

## Conclusions

This population-wide study of over 1.8 million people, uses data from the real-time Covid-19 monitoring framework in Region Stockholm. We present age stratified data for men and women, generating insights into risk factors associated with mortality, hospitalization and ICU admission with Covid-19, that can be potentially useful for guiding preventive efforts. Striking differences in risks of mortality and hospital admission with regards to co-morbities and possible co-medication raise important questions. These, and other issues will be addressed as the monitoring framework continuously is extended and updated within the Stockholm Region.

## Supplementary Information

Below is the link to the electronic supplementary material.

Supplementary file1 (DOCX 372 KB)
