# Supplementary material for: Risk factors for COVID-19-related death, hospitalization and intensive care: a population-wide study of all inhabitants in Stockholm
Source: Eur J Epidemiol. 2022 Jan 27;37(2):157–65. doi: 10.1007/s10654-021-00840-7 (PMC8791671; doi:10.1007/s10654-021-00840-7)
Supplement: Supplementary file 1 — Supplementary file1 (DOCX 372 KB) [file 10654_2021_840_MOESM1_ESM.docx]

# **Supplementary tables**

| **Table S1. List of ICD-10 codes used to define potential risk factors when extracting data from the Stockholm County central healthcare utilization data warehouse (VAL)** | |
| --- | --- |
| **Risk factor** | **ICD-10 code** |
| Hypertension | I10 |
| Heart failure | I50, I110, I43, I42 (excluding I42.1 and I42.2) |
| Ischemic Heart Disease | I20-I25 |
| Atrial fibrillation | I48 |
| Obesity | E66 |
| Diabetes type II | E11 |
| Kidney failure | N17, N18, N19 and Z491 |
|  | |

| **Table S2. Bias-minimized models for each of the studied potential risk factors for Covid-19 outcomes** | |
| --- | --- |
| **Risk factor** | **Adjustment variables** |
| Hypertension | Age, sex, COPD*, diabetes type II, kidney failure, obesity, socioeconomic status (as stratification variable) |
| Heart failure | Age, sex, COPD*, diabetes type II, hypertension, kidney failure, obesity, socioeconomic status (as stratification variable) |
| Ischemic Heart Disease | Age, sex, obesity, socioeconomic status (as stratification variable) |
| Atrial fibrillation | Age, sex, COPD*, diabetes type II, heart failure, hypertension, kidney failure, obesity, socioeconomic status (as stratification variable) |
| Obesity | Age, sex, socioeconomic status (as stratification variable) |
| Diabetes type II | Age, sex, obesity, socioeconomic status (as stratification variable) |
| Kidney failure | Age, sex, COPD*, diabetes type II, heart failure, hypertension, obesity, socioeconomic status (as stratification variable) |

COPD, Chronic Obstructive Pulmonary Disease

**Table S3. Associations of potential risk factors with Covid-19 outcomes**

| **Risk factor** | **Primary outcome:**  **Mortality with Covid-19** | | **Secondary outcome:**  **Inpatient hospitalization with Covid-19** | | **Tertiary outcome:**  **ICU admission with Covid-19** | |
| --- | --- | --- | --- | --- | --- | --- |
|  | **HR (CI)** | | **HR (CI)** | | **HR (CI)** | |
|  | **Women** | **Men** | **Women** | **Men** | **Women** | **Men** |
| **Hypertension** |  |  |  |  |  |  |
| Total | 0.87 (0.78-0.98) | 1.02 (0.92-1.14) | 1.23 (1.14-1.32) | 1.17 (1.10-1.25) | 1.69 (1.30-2.18) | 1.58 (1.36-1.84) |
| Age=60 | 0.94 (0.68-1.29) | 1.04 (0.81-1.33) | 0.99 (0.89-1.10) | 1.10 (1.01-1.20) | 1.85 (1.36-2.51) | 1.74 (1.45-2.09) |
| Age=70 | 0.87 (0.70-1.09) | 1.03 (0.87-1.22) | 1.13 (1.04-1.22) | 1.10 (1.03-1.18) | 1.43 (1.10-1.86) | 1.34 (1.13-1.58) |
| Age=80 | 0.86 (0.75-0.98) | 0.99 (0.88-1.10) | 1.37 (1.26-1.50) | 1.16 (1.07-1.26) | 1.10 (0.76-1.60) | 1.01 (0.77-1.32) |
| **Heart failure** |  |  |  |  |  |  |
| Total | 1.79 (1.58-2.03) | 1.59 (1.41-1.79) | 2.26 (2.05-2.48) | 1.70 (1.56-1.86) | 1.52 (0.98-2.35) | 1.18 (0.89-1.56) |
| Age=60 | 6.62 (4.35-10.08) | 2.00(1.29-3.12) | 2.04 (1.61-2.59) | 1.38 (1.16-1.65) | 1.58 (0.58-4.26) | 1.62 (1.08-2.42) |
| Age=70 | 4.01 (2.97-5.42) | 1.90 (1.50-2.40) | 2.08 (1.76-2.45) | 1.59 (1.40-1.80) | 2.83 (1.60-4.99) | 1.27 (0.94-1.72) |
| Age=80 | 2.61 (2.20-3.10) | 1.71 (1.49-1.95) | 2.22 (2.01-2.46) | 1.80 (1.65-1.97) | 1.91 (1.15-3.14) | 0.99 (0.65-1.49) |
| **IHD** |  |  |  |  |  |  |
| Total | 1.49 (1.30-1.71) | 1.45 (1.30-1.62) | 1.88 (1.7-2.07) | 1.62 (1.51-1.75) | 1.32 (0.82-2.12) | 1.42 (1.15-1.75) |
| Age=60 | 1.87 (0.27-13.06) | 2.20 (1.46-3.33) | 2.01 (1.62-2.51) | 1.51 (1.33-1.72) | 2.62 (1.25-5.48) | 2.08 (1.55-2.79) |
| Age=70 | 3.35 (2.44-4.60) | 2.05 (1.68-2.50) | 1.89 (1.62-2.20) | 1.52 (1.39-1.67) | 1.90 (1.07-3.39) | 1.64 (1.29-2.08) |
| Age=80 | 2.19 (1.82-2.64) | 1.59 (1.41-1.79) | 1.87 (1.69-2.07) | 1.65 (1.53-1.79) | 1.05 (0.59-1.90) | 1.00 (0.72-1.39) |
| **Atrial** **fibrillation** |  |  |  |  |  |  |
| Total | 1.07 (0.94-1.21) | 1.09 (0.97-1.23) | 1.26 (1.14-1.39) | 1.09 (1.00-1.18) | 1.16 (0.75-1.80) | 0.85 (0.65-1.11) |
| Age=60 | 0.84 (0.13-5.31) | 1.06 (0.70-1.60) | 1.11 (0.86-1.43) | 0.91 (0.76-1.08) | 1.53 (0.72-3.26) | 1.22 (0.82-1.83) |
| Age=70 | 1.33 (0.96-1.86) | 1.03 (0.82-1.30) | 1.10 (0.93-1.31) | 0.97 (0.86-1.09) | 1.29 (0.79-2.13) | 1.00 (0.74-1.33) |
| Age=80 | 1.19 (0.98-1.44) | 1.07 (0.94-1.22) | 1.22 (1.10-1.35) | 1.12 (1.02-1.22) | 1.19 (0.73-1.94) | 0.74 (0.51-1.07) |
| **Obesity** |  |  |  |  |  |  |
| Total | 2.51 (2.02-3.11) | 2.22 (1.81-2.73) | 2.74 (2.51-2.99) | 2.36 (2.15-2.58) | 3.24 (2.47-4.25) | 2.67 (2.16-3.29) |
| Age=60 | 2.79 (1.75-4.44) | 3.75 (2.63-5.34) | 3.21 (2.84-3.62) | 2.42 (2.15-2.72) | 3.58 (2.55-5.02) | 2.55 (1.97-3.30) |
| Age=70 | 2.54 (1.85-3.49) | 2.60 (2.05-3.31) | 2.56 (2.31-2.84) | 2.05 (1.84-2.27) | 2.67 (1.84-3.88) | 2.30 (1.76-3.00) |
| Age=80 | 2.43 (1.95-3.03) | 1.81 (1.41-2.34) | 1.99 (1.71-2.32) | 1.77 (1.49-2.09) | 1.92 (1.02-3.62) | 2.15 (1.34-3.46) |
| **Diabetes type II** |  |  |  |  |  |  |
| Total | 1.82 (1.61-2.05) | 1.86 (1.67-2.06) | 2.11 (1.96-2.27) | 1.94 (1.83-2.06) | 2.96 (2.29-3.83) | 2.31 (1.98-2.69) |
| Age=60 | 3.37 (2.08-5.47) | 3.59 (2.84-4.55) | 2.45 (2.16-2.78) | 2.16 (1.97-2.36) | 4.62 (3.31-6.45) | 3.18 (2.61-3.86) |
| Age=70 | 3.17 (2.48-4.05) | 2.61 (2.22-3.07) | 2.18 (1.99-2.38) | 1.90 (1.78-2.02) | 2.75 (2.08-3.64) | 2.13 (1.80-2.52) |
| Age=80 | 2.24 (1.94-2.58) | 1.90 (1.71-2.11) | 1.99 (1.83-2.16) | 1.73 (1.59-1.87) | 1.55 (1.01-2.36) | 1.31 (0.98-1.73) |
| **Kidney failure** |  |  |  |  |  |  |
| Total | 1.35 (1.18-1.55) | 1.85 (1.65-2.08) | 1.56 (1.41-1.73) | 1.86 (1.71-2.02) | 2.33 (1.58-3.45) | 1.26 (0.96-1.65) |
| Age=60 | 5.17 (3.26-8.2) | 4.05 (2.88-5.68) | 2.74 (2.18-3.44) | 2.21 (1.89-2.59) | 6.54 (3.82-11.17) | 1.92 (1.32-2.80) |
| Age=70 | 3.34 (2.42-4.62) | 3.07 (2.47-3.82) | 2.18 (1.86-2.56) | 2.00 (1.79-2.23) | 3.26 (2.13-4.99) | 1.39 (1.04-1.86) |
| Age=80 | 2.05 (1.7-2.46) | 2.18 (1.92-2.47) | 1.66 (1.49-1.84) | 1.81 (1.66-1.98) | 1.34 (0.74-2.43) | 0.97 (0.65-1.46) |

Adjusted hazard ratios (HR) and 95% confidence intervals (CI) for the association of each potential risk factor with each outcome. Covariates included in the analyses were guided by the causal diagram presented in Supplementary Table S2.

# **Supplementary figures**

**Figure S1 and S2 Epi-curves of all Covid-19 cases and Covid-19-related deaths in Stockholm County during the study period (March 1^st^, 2020 to July 31^st^, 2020)**

**Figure S3.** **Study population flow chart**

# **Figure S4. Causal diagram for hypertension as a risk factor for Covid-19 outcomes**

COPD, Chronic Obstructive Pulmonary Disease

# **Figure S5. Causal diagram for heart failure as a risk factor for Covid-19 outcomes**

COPD, Chronic Obstructive Pulmonary Disease

# **Figure S6. Causal diagram for ischemic heart disease (IHD) as a risk factor for Covid-19 outcomes**

COPD, Chronic Obstructive Pulmonary Disease

# **Figure S7. Causal diagram for atrial fibrillation as a risk factor for Covid-19 outcomes**

COPD, Chronic Obstructive Pulmonary Disease

# **Figure S8. Causal diagram for obesity as a risk factor for Covid-19 outcomes**

COPD, Chronic Obstructive Pulmonary Disease

# **Figure S9. Causal diagram for diabetes type II as a risk factor for Covid-19 outcomes**

COPD, Chronic Obstructive Pulmonary Disease

# **Figure S10. Causal diagram for renal failure as a risk factor for Covid-19 outcomes**

COPD, Chronic Obstructive Pulmonary Disease
